# Supplementary material for: Reconstructing disease dynamics for mechanistic insights and clinical benefit
Source: Nat Commun. 2023 Oct 27;14:6840. doi: 10.1038/s41467-023-42354-8 (PMC10611752; doi:10.1038/s41467-023-42354-8)
Supplement: Supplementary file 1 — Supplementary Information [file 41467_2023_42354_MOESM1_ESM.pdf]

Supplementary Note 1

The TimeAx algorithm can be divided into three steps: conserved-dynamics-seed selection, multiple trajectory alignment and disease pseudotime estimation (**Figure S1A**).

Step 1 (conserved-dynamics-seed selection)

Identifying the consensus of a disease requires common ground. We thus start off by choosing a set of *"conserved-dynamics-seed" features (seed -features).* These features can be predefined by the user or can be computationally selected by focusing on features *whose* dynamics are similar across individuals. Features are scaled into [0,1] range by subtracting their minimal value and dividing by the maximal value minus the minimal value. To avoid overfitting, TimeAx focuses on those features with high variation across the profiles. Therefore, it requires seed features to have many unique values (different values in most of the measured profiles) and a high standard deviation across profiles, both of which must be above user determined thresholds. Next, for each feature, TimeAx applies an iterative bootstrap-like process (20 iterations by default), where in each iteration it samples an equal number of time points from each pair of patients and calculates their spearman correlation for the feature's expression across these time points. The number of time points sampled equals a percent (80% by default) of the time points of the patient with the least amount of time points. Following this, TimeAx averages correlation values across all pairs of patients and iterations and chooses *L* (user-defined; default of 50) features with the highest values as being part of the "*conserved-dynamics-seed*". Alternatively, the "*conserved-dynamics-seed*" can be pre-defined as an input by the user, disabling the computational selection of seed features.

Step 2 (multiple trajectory alignment)

To assemble a consensus trajectory describing the complete disease dynamics, we rely on principles stemming from the mature field of DNA sequence alignment. Specifically, in progressive multiple sequence alignment (MSA) methods, DNA sequences are added into the MSA in an orderly fashion based on a guide tree, structured according to the pairwise alignment distance of all sequences [1]. Similarly, as described below, TimeAx performs a multiple trajectory alignment (MTA) on time-series datasets of individuals each of which we consider as an individual partial trajectory. The alignment process relies solely on the above described "*conserved-dynamics-seed*" (Step 1).

In TimeAx, the leaves of the guide tree represent the original individual subject partial trajectories, whereas the inner nodes of the tree represent pseudo-trajectories created based on different combinations of the leaves' trajectories. During the multiple alignment step, TimeAx follows the structure of the tree and merges each two child nodes into a new parent node. Specifically, given trajectories 1 and 2, TimeAx leverages dynamic time warping [2,3] to detect the best fit between sample profiles in trajectory 1 and in trajectory 2, keeping the order of both trajectories. Based on this pairwise alignment, TimeAx yields a new, intermediate trajectory with a confidence score, calculated as the average of correlation

coefficients between the aligned profiles that were used in its creation. Next, TimeAx calculates new intermediate profiles as the average between the aligned profiles, weighted by their confidence scores. The confidence scores of the initial trajectories (corresponding to different individuals) are the average of the confidence scores obtained by aligning them with all the other trajectories. At the end of the process, the final node, located at the root of the guide tree is the consensus trajectory.

<u>Pseudocode:</u>
Let *trajectories* be a set of *n* trajectories, each represents a time series data from an individual.

Create a random guide tree (T) for all trajectories

Initiate confidence scores for all trajectories ($Conf_i \ldots Conf_n$)

While size of T > 1:

    Choose two leaves i and j

    $\mathrm{Align_{ij}} = $ a DTW alignment between $\mathrm{Traj_i}$ and $\mathrm{Traj_j}$

    Create a new parent node z

    Based on $\mathrm{Align_{ij}}$, calculate $\mathrm{Conf_z}$

    $\mathrm{Traj_z} = \mathrm{Traj_i} \cdot \mathrm{Conf_i} + \mathrm{Traj_j} \cdot \mathrm{Conf_j}$

    Remove nodes i and j from the tree

$\mathrm{Traj_{root}} = $ consensus trajectory

To obtain a continuous measure of progression across the consensus trajectory, TimeAx then calculates numeric disease pseudotime positions for each of the consensus trajectory profiles. To do so, we apply a principal component analysis (PCA) to the profiles (using only the seed features), and, beginning from PC1, the set of PCs that sum up to 90% of the variance are selected. The sum of distances across all selected PCs are then calculated and transformed into a [0,1] scale, by removing their minimal value and dividing by the maximal value minus the minimal value.

One of the obstacles of MSA is the selection of a good guide tree. As the structure of the guide tree determines the alignment ordering, it has a strong effect on the resulting consensus trajectory. To overcome this, TimeAx takes an ensemble approach, creating N random guide trees (along this manuscript we use *N*=100 by default), representing a set of weak learners [4]. Therefore, while each guide tree is non-optimal, the combination of the whole set provides high accuracy levels. Based on this selection of guide trees, the final model obtained by TimeAx, contains multiple consensus trajectories which can be used to predict the disease

pseudotime of the original samples across individuals, as well as allow the disease pseudotime prediction for new samples (Step 3).

Step 3 (disease pseudotime estimation)

TimeAx allows the prediction of disease pseudotime for new samples, based on the set of consensus trajectories, generated during the alignment process (Step 2). To overcome technical differences between the consensus profiles, devised based on the train longitudinal data, and the new samples, TimeAx allows the prediction to be based on the ratios between features rather than the features themselves. Regardless, TimeAx first normalizes the values of each feature within the consensus profiles and uses the normalizing factors (mean and standard deviation) to transform the new samples as well. Next, TimeAx calculates the spearman correlation between each new sample normalized profile and the normalized profiles of all states within the consensus trajectory and chooses the highest correlated state as its predicted local disease pseudotime position. Finally, as being an ensemble method, TimeAx calculates the disease pseudotime position by averaging the predictions across all guide trees. TimeAx also provides an uncertainty score for each sample profile, by calculating the standard deviation across the predicted disease pseudotime positions. By definition, low value of uncertainty implies a high concordance between disease pseudotime positions across all consensus trajectories.

Supplementary Note 2

We devised a robustness score to assess the success of capturing a meaningful dynamics of the disease modeled by TimeAx, under the assumption that on average the disease dynamics in patients progresses towards some end goal (e.g. disease resolution, progressive disease, or some new homeostatic state). The robustness score reflects the level of agreement between TimeAx derived disease pseudotime and a disease pseudotime, assembled with a constraint that the disease pseudotime for consecutive samples within the same individual cannot be lower than previous disease pseudotime (see below). Assuming the TimeAx model is robust, consecutive profiles should on average advance along the consensus trajectory, and therefore should provide similar predictions to the constrained model, even though sample ordering was not provided as input. On the other hand, if predictions largely differ, it implies that TimeAx failed modeling the change in condition over time (or that the disease does not obey the underlying disease progression assumption TimeAx relies on). In that case, the model robustness will be low.

To derive the robustness disease pseudotime from the consensus trajectory, for a new individual with L profiles, TimeAx first calculates distance matrix D, as one minus the spearman correlation between its profiles and the M profiles of a consensus. The distances are then smoothed to increase prediction robustness (smoothing as in CellAlign interpolation [2]). Next TimeAx constructs a weighted directed graph with L layers and M nodes in each layer. Each node in layer i is connected to nodes in layer $i+1$ that represent profiles located in the same level or higher in the consensus trajectory. The weight of an edge that is directed to node j in layer i is D_{ij} $[i \in \{1 \dots L\} \& j \in \{1 \dots M\}]$. In addition, a "start" node is directed to all nodes in the first layer and all nodes in layer L are directed to an "end" node (**Figure S1B**). Finally, Dijkstra's algorithm is used to calculate the shortest path between "start" and "end", pointing out the best set of states in the consensus fitting to the profiles of the new individual. To obtain the final robustness disease pseudotime, the results across all different computed consensus trajectories are then averaged. The robustness score is calculated as the Pearson correlation coefficient between disease pseudotime and robustness disease pseudotime.

Supplementary Note 3

To test TimeAx's ability to capture the dynamics of a disease, we first performed a simulation study. The framework consists of two main parts (**Figure S1C**):

1. Generation of synthetic dataset.
2. Method comparison.

Generation of synthetic dataset:
For each combination of simulation parameters (See **Supplementary Table 1**), we created a set of synthetic datasets, each with a unique pseudotime axis (ranging from 0 to 1), representing the real dynamics of the simulated disease. In each dataset, we generated N subjects, each containing *M* consecutive samples. For each subject, we chose a starting and an achieved position along the generated pseudotime and picked *m* random points in between, corresponding to samples. We denoted the pseudotime positions that correspond to the selected samples as *"True pseudotime"*, whereas the *"Chronological time points",* are the samples' order within each individual (1…M).

For each dataset, we generated an expression for *K* genes, where each gene had a unique pattern along the *True pseudotime* in one of three pattern types:

1. Significant linear upregulation/downregulation ("linear pattern");
2. Significant quadratic behavior ("non-linear pattern");
3. Insignificant change ("no pattern").

Therefore, gene expression in pseudotime position 0 represents the beginning of the gene's pattern while 1 represents its end. Since time points for each subject were chosen at different ranges across the *True pseudotime* axis, chronological time points corresponded to different gene expression states for each subject (**Figure S1D**). Expression levels were modeled with a gamma distribution with shape and scale parameters chosen to maintain a predefined coefficient of variation. Specifically, coefficients of variations were calculated as the ratio between a standard deviation and a mean value of expression. The set of main datasets' parameters is given in **Supplementary Table 1**.

Supplementary Table 1

	**Parameter**	**Description**
Variable parameters	Number of subjects (N)	10..100 (20 by default)
	Number of time points per subject (M)	3..10 (5 by default)
	Noise coefficient of variation (CV)	0..0,1 (0.025 by default)
Fixed parameters	Fraction of significant genes	20%
	Fraction of significant features with non-linear expression pattern	50%

	Number of features (K)	15k
	Number of simulations per combination	40

Method comparison:

Based on the simulated datasets, we then compared the efficiency of three different longitudinal frameworks for the estimation of *True pseudotime* and the detection of dynamics' significant genes:

1. TimeAx
2. Diffusion maps (DMs)
3. Chronological time

While TimeAx and DMs generated estimated pseudotime based on the generated expression datasets, in chronological time, the estimated pseudotime was equal to the generated chronological time points. Then, we inferred significant genes by performing a quadratic regression between the estimated pseudotime and each gene's expression across all samples from all subjects. Genes with false discovery rate (FDR) corrected p-values below 0.05 were selected as significant. Finally, we used Spearman correlation between the estimated pseudotime and True pseudotime as a measure of pseudotime estimation accuracy and Area Under Curve (AUC) between the priorly selected significant genes and the discovered genes as a measure of gene detection accuracy.

TimeAx displayed high accuracy in discovering significant genes, compared to DMs and chronological time, while also allowing the estimation of True pseudotime, which was largely missed by the alternative approaches. TimeAx's superior accuracy was observed across different coefficients of variation (**Figure S1E**), using different amounts of time points per subject (**Figure S1F**) and for a variety of cohort sizes (**Figure S1G**).

Supplementary Note 4 - TimeAx deciphers AMD disease dynamics from longitudinal monitoring of clinical imaging data

We studied how structural changes within the retina are associated with the progression of AMD. We discovered a strong positive correlation of disease pseudotime with retinal fibrosis and atrophy, as opposed to a negative correlation with retinal pigment epithelium (RPE) density, in agreement with previous findings of these structural features as hallmarks of AMD progression [5–8] (r=0.47, 0.7 and -0.68, respectively; **Figure S4B**). The progression along these structural features followed a nonlinear fashion, with a relatively low degree of change in low disease pseudotime positions, compared to a major shift at high positions, suggesting the initiation of a more aggressive state of the disease (at disease pseudotime position 0.75; **Figure S4B**). In addition, we found a peak in drusen at low disease pseudotime positions (at disease pseudotime position 0.25), corroborating drusen as an early marker of AMD [9] (**Figure S4B**). Finally, we observed a gradual decrease in the density of neurosensory nerves in the retina density over the disease pseudotime, pointing to a novel association with disease progression, presenting distinct densities throughout all stages of the disease (**Figure S4B**).

We wanted to showcase the utility of disease pseudotime for clinical diagnosis of AMD patients' disease states. Since the TimeAx model integrates the contributions of different structural features of the retina, we hypothesized that it will capture an improved representation of the clinical state of the patients. Indeed, different visual acuity levels were characterized by distinct ranges of disease pseudotime positions, while only retinal atrophy displayed distinct levels at higher visual acuity, showing no difference at earlier visual acuity levels (**Figure S4C**). This highlights the benefits of monitoring early disease progression stages, where disease progression can still be hindered, using disease pseudotime. In addition, by dividing patients into severity groups based on distinct levels of features, we found the less false positive classifications, based on disease pseudotime, supporting its utility for patient diagnosis and monitoring (**Figure S4D**).

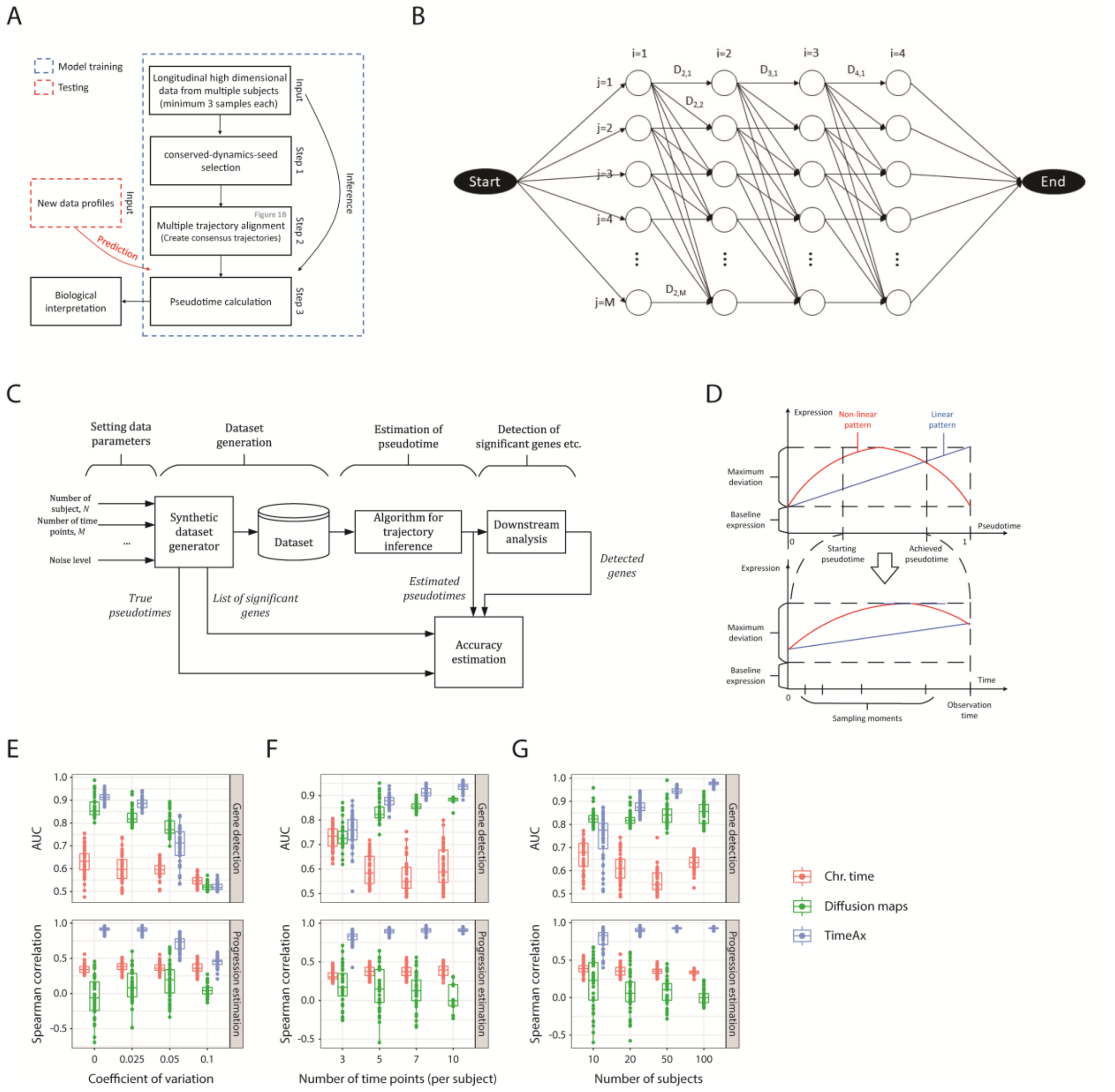

Figure S1: A. A workflow diagram of TimeAx. **B.** A representative graph for calculating the disease robustness pseudotime for an individual with 4 time points and a consensus trajectory with M disease pseudotime positions (See **Supplementary Note 2**). **C-G.** Simulated data analysis. **C.** An illustration of the simulation framework. **D.** Gene generation pipeline and sample selection based on either linear or quadratic trend over true pseudotime. **E-G.** Superior results for TimeAx predicted disease pseudotime (blue), compared to simulated subjects' time points (Chr. time; red) and diffusion maps (green), in capturing significant genes (top) and the the values of the true pseudotime (bottom) across different levels of coefficients of variation **(E)**, number of samples for each subject **(F)** and the number of subjects **(G)** (*x*-axis). Disease pseudotime prediction accuracy was calculated using the Spearman correlation with true disease pseudotime, while significant gene detection accuracy was inferred using the AUC score (See **Supplementary Note 3**). In **E-G**, boxes represent the 25th, 50th, and 75th percentiles; whiskers show maxima and minima and *n*=40 simulation results per box. Area Under Curve (AUC).

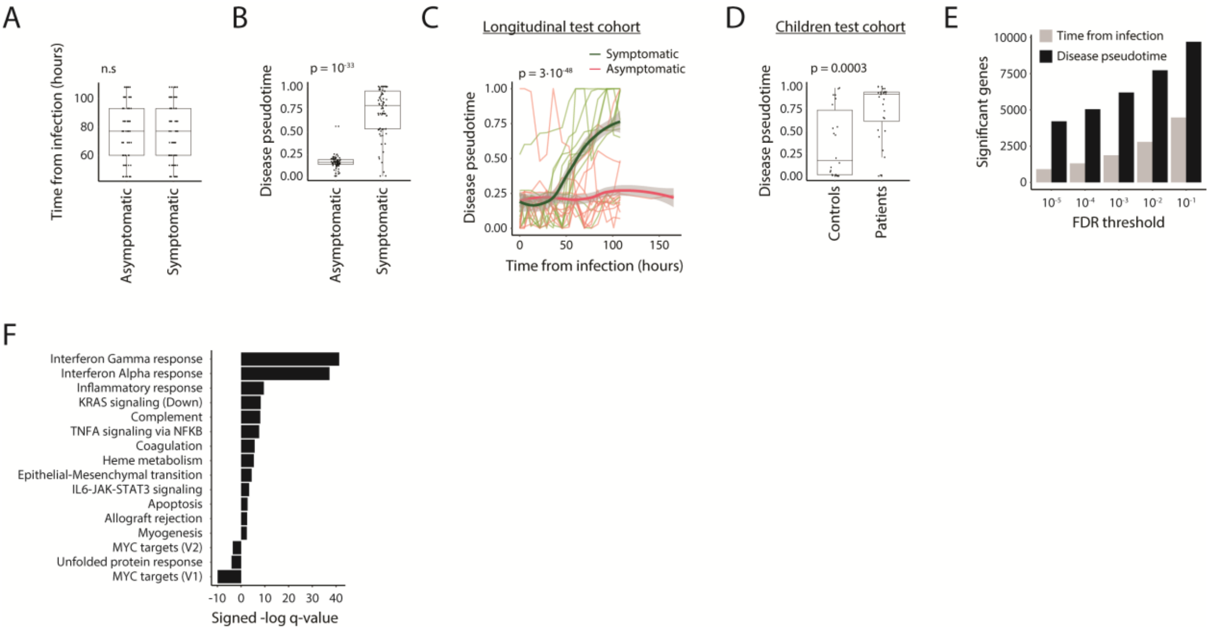

Figure S2: A. No significant difference in chronological time after infection (*y*-axis) between symptomatic and asymptomatic patients, in late time points after infection (>45 hours). **B-C.** Significantly higher disease pseudotime positions (*y*-axis) for symptomatic patients compared to asymptomatic patients, in late time points after infection (>45 hours), using the longitudinal train cohort (**B**) and the longitudinal test cohort (**C**). In **C,** p-value was calculated by comparing the prediction of disease pseudotime (by ANOVA), using only time or the interaction between time and symptoms as predictors. Trend lines represent the average levels in each of the groups +/- standard error). **D.** Significantly higher disease pseudotime positions (*y*-axis) for patients compared to healthy controls, using the children test cohort. **E.** Number of progression-related genes (*y*-axis), across different *q*-value thresholds (*x*-axis), is higher using pseudotime (black), compared to sampling time (gray), for the longitudinal train cohort (See **Methods**). **F.** Enrichment scores of biological pathways from MSigDB Hallmarks, based on associations of genes with the disease pseudotime in the longitudinal train cohort (See **Methods**). In **A, B** and **D**, boxes represent the 25th, 50th, and 75th percentiles; whiskers show maxima and minima (**A**,**B**: *n*=268, **D**: *n*=101). P-values were calculated based on a two-sided t-test.

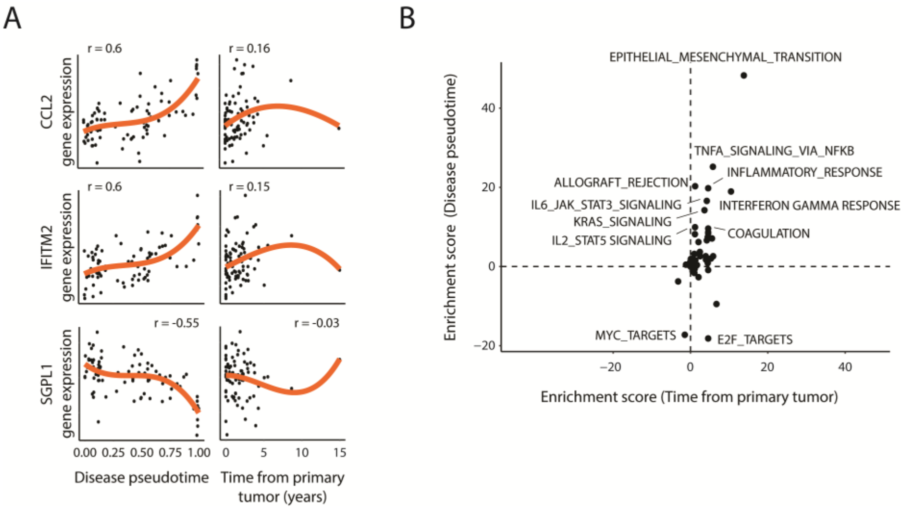

Figure S3: A. *CCL2*, *IFITM2* and *SGPL1* expression levels (*y*-axis) along either disease pseudotime (left; strong associations) or tumor recurrence from primary tumor (right; weak associations) (*x*-axis). Regression trend line is displayed in orange. **B.** Enrichment scores of biological pathways from MSigDB Hallmarks (black dots), displaying improved scores while calculated using pseudotime (*y*-axis) compared to tumor recurrence times (*x*-axis).

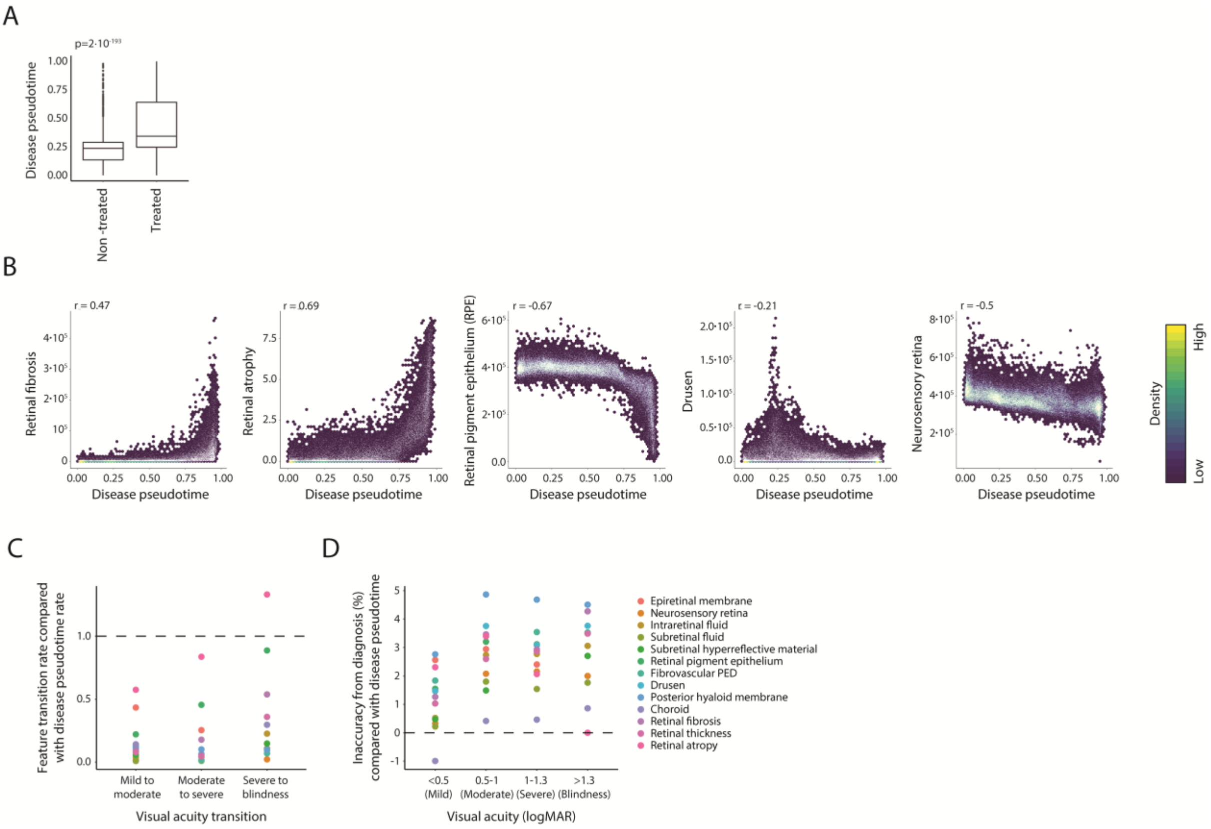

Figure S4: A. Disease pseudotime (*y*-axis) distribution in anti-VEGF treated and non-treated patients (*x*-axis) from the AMD test cohort (*n*=29205 biologically independent samples). Boxes represent the 25th, 50th, and 75th percentiles; whiskers show maxima and minima. P-value was calculated based on a two-sided t-test. **B.** Associations of different segmented structural features of the retina (*y*-axis) with the disease pseudotime (*x*-axis), across all data points from the AMD test cohort. **C.** Ratios between segmented features' (color-coded) and disease pseudotime's average transition rates (*y*-axis), across each pair of consecutive visual acuity clinical states (*x*-axis) (See **Methods**). **D.** Percentage difference between segmented features' (color-coded) and disease pseudotime's error rates (*y*-axis), within each visual acuity clinical state (*x*-axis) (See **Methods**).

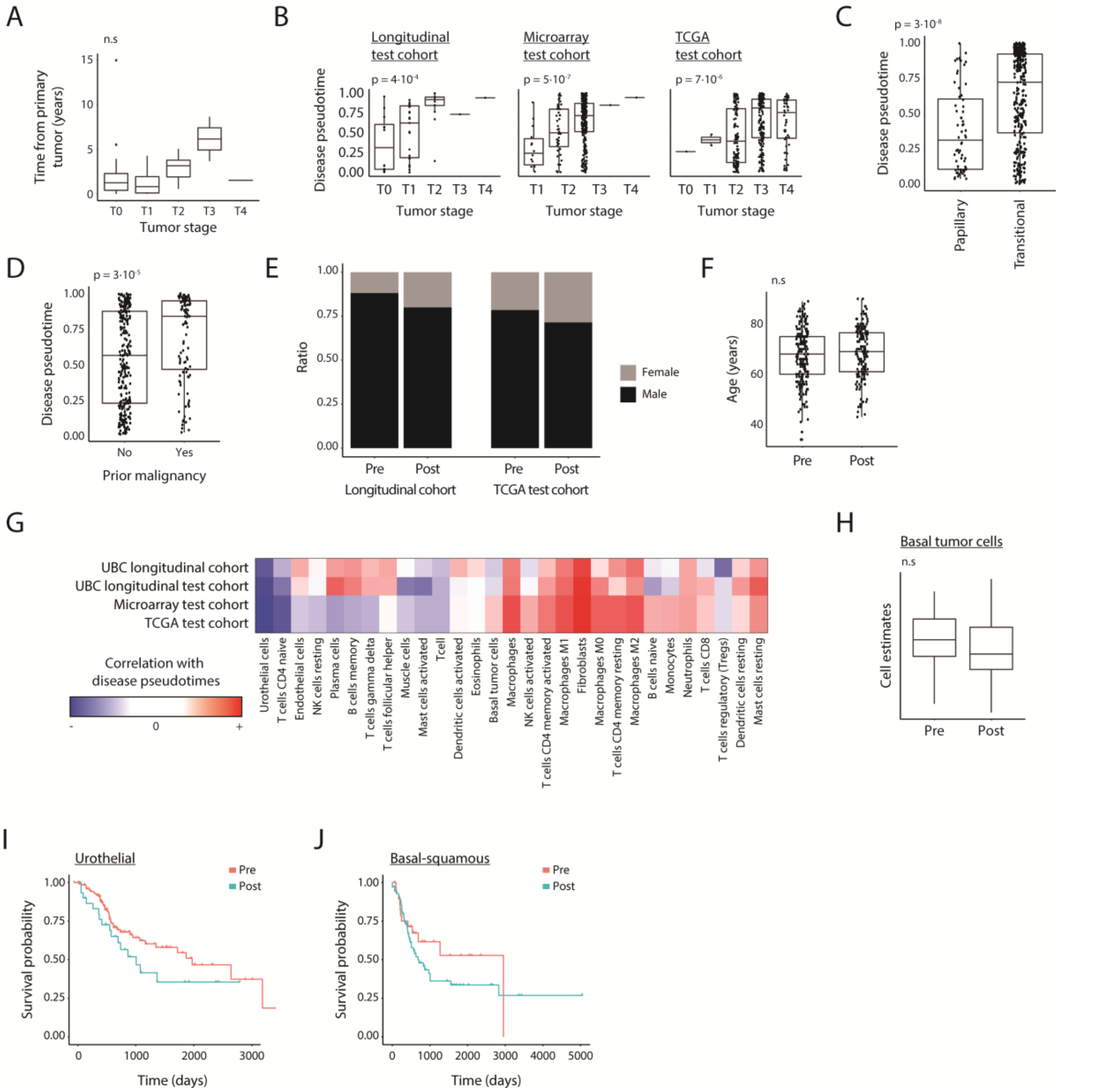

Figure S5: A. Chronological time (time from primary tumor diagnosis; *y*-axis) across tumor stages (*x*-axis) for the longitudinal cohort. **B**. Shown are disease pseudotime (*y*-axis) relations with tumor stage (*x*-axis) for the longitudinal (left), microarray (middle) and TCGA (right) test cohorts. **C.** Disease pseudotime (*y*-axis) is significantly higher in transitional compared to papillary tumors. **D.** Disease pseudotime (*y*-axis) is significantly higher in patients with prior malignancies. **E-F.** Lack of association for patients' Sex (**E**) and Age (**F**) with division into pre- vs. post- stromal pro-invasion point (*x*-axis). **G.** Pearson correlations of deconvolved cell compositions with disease pseudotime, across all samples in both the UBC longitudinal train cohort as well as the longitudinal test, microarray and TCGA test cohorts (See **Methods**). Negative to positive correlations are colored in a blue to red color scale. **H.** Basal tumor cells deconvolved composition (*y*-axis) in pre- and post- stromal pro-invasion point samples (*x*-axis), presenting no significant difference between the two. **I-J**. Survival plots for either Urothelial-like (**I**) or Basal-squamous (**J**) patients within the TCGA validation cohort, comparing patients' tumors with disease pseudotime positions lower and higher the stromal pro-invasion point (pre vs. post, respectively; color coded). In **A-D, F** and **H**, boxes represent

the 25th, 50th, and 75th percentiles; whiskers show maxima and minima. For **A**,**B**,**C**,**D**,**F**,**H** *n*=84,52,382,382,382,84, respectively. In **A**,**B** P-values based on linear regression. In **C**,**D**,**F**,**H** P-values were calculated based on a two-sided t-test.

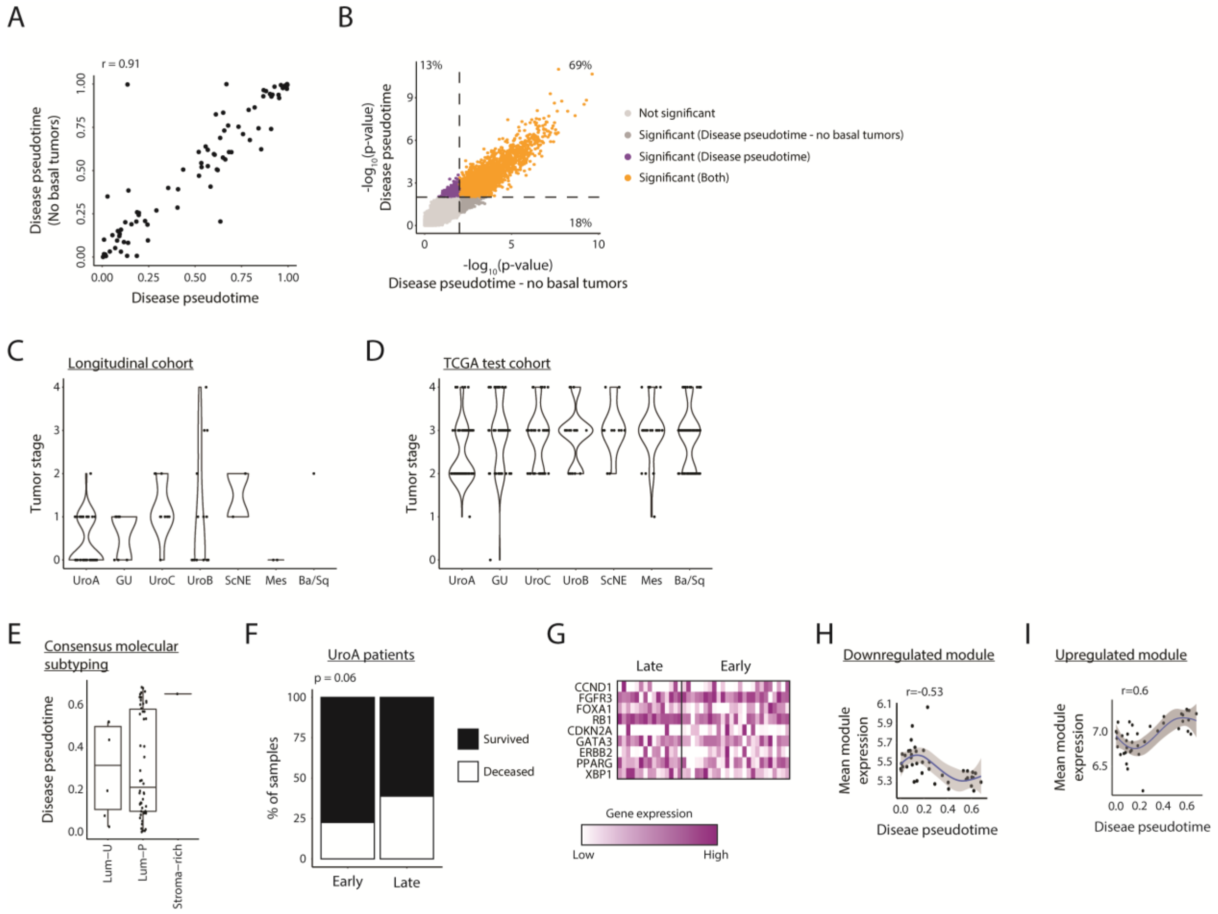

Figure S6: A. Strong association between disease pseudotime inferred by model using all patients (*x*-axis) and all patients including only non-basal tumors (excluding basal tumors while TimeAx modeling; *y*-axis) from the UBC longitudinal cohort. **B.** Gene associations (-log10 transformed, P-values based on linear regression) with disease pseudotime inferred by model using all patients (*x*-axis) or all patients excluding basal tumors (*y*-axis), using a *p*-value threshold of 10^{-2} (Dashed lines) (See **Methods**). Genes are colored based on their association with the two time axes. **C-D.** Lack of association between tumor stages (*y*-axis) and tumor molecular classifications in patients within the UBC longitudinal cohort (**C**) and the TCGA test cohort (**D**) based on the 'LundTax' tumor molecular classification framework. (**E**) Disease pseudotime positions (y-axis) of Lum-P, Lum-U and Stroma Rich molecular types tumors (classified by the consensus molecular subtyping, X axis). **F.** Survival (black) versus deceased (white) percentages of UroA patients within the TCGA test cohort at the early and late patient groups (*x*-axis). p-value was calculated using Fisher's exact test. **G.** Heatmap of the expression levels of UroA marker genes (rows), in early and late UroA tumors (columns) within the UBC longitudinal cohort. **H-I.** Average expression levels of genes within the downregulated **(H)** and upregulated **(I)** modules (*y*-axis) along the disease pseudotime (*x*-axis) in patients pre-stromal pro-invasion point. Trend lines represent the average across points along the disease pseudotime +/- standard error.

Supplementary References

1. Feng, D. F. & Doolittle, R. F. Progressive sequence alignment as a prerequisite to correct phylogenetic trees. *J. Mol. Evol.* **25**, 351–360 (1987).
2. Alpert, A., Moore, L. S., Dubovik, T. & Shen-Orr, S. S. Alignment of single-cell trajectories to compare cellular expression dynamics. *Nat. Methods* **15**, 267–270 (2018).
3. Vintsyuk, T. K. Speech discrimination by dynamic programming. *Cybern. Syst. Anal.* **4**, 52–57 (1968).
4. Tin Kam Ho. Random decision forests. in *Proceedings of 3rd International Conference on Document Analysis and Recognition* 278–282 (IEEE Comput. Soc. Press, 1995). doi:10.1109/ICDAR.1995.598994.
5. Cheung, C. M. G. *et al.* The Evolution of Fibrosis and Atrophy and Their Relationship with Visual Outcomes in Asian Persons with Neovascular Age-Related Macular Degeneration. *Ophthalmol. Retina* **3**, 1045–1055 (2019).
6. Miere, A. *et al.* Optical coherence tomography angiography features of subretinal fibrosis in age-related macular degeneration. *Retina (Philadelphia, Pa)* **35**, 2275–2284 (2015).
7. Fleckenstein, M. *et al.* The Progression of Geographic Atrophy Secondary to Age-Related Macular Degeneration. *Ophthalmology* **125**, 369–390 (2018).
8. Kinnunen, K., Petrovski, G., Moe, M. C., Berta, A. & Kaarniranta, K. Molecular mechanisms of retinal pigment epithelium damage and development of age-related macular degeneration. *Acta Ophthalmol.* **90**, 299–309 (2012).
9. Flores, R., Carneiro, Â., Tenreiro, S. & Seabra, M. C. Retinal Progression Biomarkers of Early and Intermediate Age-Related Macular Degeneration. *Life (Basel)* **12**, (2021).
