# Supplementary material for: Reconstructing disease dynamics for mechanistic insights and clinical benefit
Source: Nat Commun. 2023 Oct 27;14:6840. doi: 10.1038/s41467-023-42354-8 (PMC10611752; doi:10.1038/s41467-023-42354-8)
Supplement: Supplementary file 3 — Description of Additional Supplementary Files [file 41467_2023_42354_MOESM3_ESM.pdf]

Description of Additional Supplementary Files

File name: Supplementary Data 1

Description: Statistics of the Cox proportional-hazards model classifying patients survival rates for either pre- or post- stromal pro-invasion point tumors, after accounting for other covariates with known association with survival, including age, sex and the clinical stage of the disease.

File name: Supplementary Data 2

Description: Differential expression of genes (column 1) between UroA early tumors (mean expression in column 2) and UroA late tumors (mean expression in column 3). Significance value, based on a two-sided student's t-test is presented in column 4.

File name: Supplementary Data 3

Description: Highly enriched pseudogene and microRNAs groups within the downregulated module (column 1), their number of appearances in the module (column 2) and their equivalent enrichment score (*q*-value) within the module, based on an upper tail hypergeometric test (column 3).

File name: Supplementary Data 4

Description: Highly enriched gene sets related to membrane channel proteins and GPCRs within the downregulated module (column 1), their number of appearances in the module (column 2) and their equivalent enrichment score (*q*-value) within the module, based on an upper tail hypergeometric test (column 3).

File name: Supplementary Data 5

Description: Highly enriched gene sets within the upregulated module (column 1), their number of appearances in the module (column 2) and their equivalent enrichment score (*q*-value) within the module, based on an upper tail hypergeometric test (column 3).
